# Fostering voice behavior in correctional institutions: Investigating the role of organizational support and proactive personality

**DOI:** 10.1371/journal.pone.0303768

**Published:** 2024-05-17

**Authors:** Dodot Adikoeswanto, Siti Nurjanah, Saparuddin Mukhtar, Anis Eliyana, Andika Setia Pratama, Rachmawati Dewi Anggraini, Nurul Liyana Mohd Kamil

**Affiliations:** 1 Postgraduate School, Universitas Negeri Jakarta, East Jakarta, Indonesia; 2 Directorate General of Corrections, Ministry of Law and Human Rights of the Republic of Indonesia, Central Jakarta, Indonesia; 3 Department of Management, Universitas Airlangga, Surabaya, Indonesia; 4 Department of Research and Publication, PT Usaha Mulia Digital Indonesia, South Jakarta, Indonesia; 5 Department of Political Sciences, Public Administration and Development Studies, Universiti Malaya, Kuala Lumpur, Malaysia; Kalasalingam Academy of Research and Education, INDIA

## Abstract

This research delves into the intricate interplay between perceived organizational support, proactive personality, and voice behavior. Furthermore, it establishes the pivotal role of work engagement as a mediating factor within the articulated research model. The study engaged 287 healthcare professionals within correctional institutions and detention centers in Indonesia, employing a dual-phase questionnaire distribution to capture the dynamic aspects of the participants’ experiences. Utilizing the statistical technique of Partial Least Square—Structural Equation Modeling with the SmartPLS 4 program as an analysis tool, the collected data underwent comprehensive analysis. The outcomes reveal that proactive personality significantly influences voice behavior both directly and indirectly through its impact on work engagement. Conversely, perceived organizational support directly influences work engagement but does not exhibit a direct impact on voice behavior. These findings underscore the significance of proactive personality in fostering a conducive environment for constructive organizational change from a grassroots perspective. The study suggests that organizations prioritize the cultivation of proactive personality traits to stimulate voice behavior, thereby facilitating ongoing improvements and sustainable organizational progress.

## 1. Introduction

Organizations inevitably confront both intentional and unforeseen changes [1]. Acknowledging that employees are not mere passive recipients of change [2], it becomes apparent that their active participation is crucial to mitigate the negative impacts of organizational changes and dynamics. In this context, employees assume the responsibility of offering specific information and proposing initiatives to alleviate uncertainty [3]. Examined through the lens of organizational behavior, voice behavior emerges as a pivotal factor in the creation and implementation of ideas, the prevention of problems, the initiation of constructive change efforts, and the active articulation of crucial information that the organization needs to be aware of [4, 5].

Within the organizational context, detention centers and correctional institutions grapple with challenges such as overcrowding [6–8] and the prevalence of health issues among residents [9]. In Indonesia, detention centers and correctional institutions, in general, contend with a shortage of health facilities, medical personnel, and healthcare workers [10]. This underscores the critical need for organizational support to promote optimal health services within these facilities. The scarcity of health resources in correctional institutions and detention centers in Indonesia emphasizes the significance of organizational support in fostering an environment conducive to the delivery of quality health services. Numerous studies attest that perceived organizational support plays a pivotal role in cultivating employee responsibility and aligning work behavior with organizational objectives [11–13]. These findings suggest that perceived organizational support becomes particularly crucial in sustaining and fortifying the efforts of medical personnel and healthcare workers when faced with limited facilities and infrastructure, thereby ensuring the provision of adequate health services for prisoners and detainees.

In addition, various studies have highlighted the importance of proactive personality in organizations that face dynamics [14–16]. This is because individuals with proactive personality can adapt to various situations and tend to do more [17]. In other words, proactive personality people will make positive situational changes in their organization [18]. Thus, this study considers that proactive medical and health workers are needed and important in meeting the need for health services in correctional institutions and detention centers to run optimally.

This research model is crafted to explore the role of perceived organizational support and proactive personality as key drivers of voice behavior. The theoretical underpinnings of this mechanism draw from two well-established theories: Organizational Support Theory (OST) [19] and Trait Activation Theory (TAT) [20]. OST, an amalgamation of social exchange theory and self-enhancement processes, elucidates how perceived organizational support fosters positive attitudes and behaviors directed towards the organization [21], with a specific focus on voice behavior in this study. Furthermore, perceived organizational support offers assistance to individuals in performing effectively and navigating challenging work situations [22]. Concurrently, TAT posits that certain traits are more likely to manifest in situations where their relevance is prominent [23]. In the context of this study, the theoretical premise suggests that individuals with proactive personalities are predisposed to exhibiting voice behavior within the workplace. This dual-theory framework provides a robust foundation for examining the intricate dynamics between perceived organizational support, proactive personality, and the manifestation of voice behavior in organizational settings.

In addition, this study also examines the role of work engagement in the proposed model. Based on social exchange theory, work engagement is an integral element of driving behavior that ensures organizational sustainability [24]. Work engagement is also a unique construct that can link individual factors such as proactive personality to various positive behaviors in organizations [16, 24, 25]. Previous research also shows that work engagement plays a mediating role in the effect of perceived organizational support on voice behavior [26].

The research landscape offers diverse perspectives on the relationship between perceived organizational support, proactive personality, and employee voice behavior. Previous study discovered that perceived organizational support does not exert a direct influence on employee voice behavior [26]. Similarly, the other study conducted revealed a weak and even statistically insignificant positive effect of subordinate proactive personality on voice behavior when examining direct influence [27]. Adding to this discourse, individuals lacking proactivity in their self-concept are less inclined to express a desire for voice [28]. These disparate findings, which deviate from established theoretical foundations and introduce inconsistency, have prompted the present study. It aims to scrutinize the nuanced effects of perceived organizational support and proactive personality on employee voice behavior, particularly within the unique context of correctional institutions and detention centers. In these specialized organizational settings, voice behavior assumes a critical role as a strategic signal in decision-making processes, particularly concerning health services—a fundamental pillar in meeting the basic needs of correctional facilities.

Based on this rationale, our study seeks to investigate how perceived organizational support and proactive personality influence employee voice behavior, with work engagement acting as a mediator. Despite the significance of these variables, empirical research examining their collective impact on employee voice behavior remains scarce. This gap is evident in the most recent systematic literature review on employee work behavior, which has overlooked perceived organizational support and work engagement as potential antecedents [29]. Furthermore, our study addresses the insights provided by another systematic literature review, highlighting the scarcity of empirical studies on employee voice behavior in public organizations and the underutilization of time-lagged research designs [30]. Consequently, this study contributes novelty both in terms of context and methodology, offering perspective that received limited attention.

This study offers valuable insights in two key areas. Firstly, it illuminates the interplay between proactive personality, perceived organizational support, and work engagement, specifically in fostering constructive organizational behavior, namely voice behavior. Secondly, it employs a two-wave time lagged distribution method, overcoming limitations associated with cross-sectional designs in previous research. This methodological improvement enhances the depth and robustness of the findings, providing a nuanced understanding of the examined relationships. Additionally, the study explores an under-researched organizational context—medical officers and health workers in Indonesian correctional institutions and detention centers. In summary, this study acts as a corroborative effort, building on insights within the limited organizational contexts studied, particularly in Southeast Asia. Its broader goal is to enhance understanding of voice behavior development within organizations and provide practical recommendations, especially for correctional institutions and detention centers. The study advocates for a bottom-up approach to improve effectiveness and facilitate continuous improvement in health services.

## 2. Literature review

### 2.1 Conceptual review

#### 2.1.1 Perceived organizational support

Perceived organizational support (POS) reflects employees’ overall perception of how much their organization values their contributions and cares about their well-being [11]. It can be understood as the assurance that the organization will provide the necessary support when employees require assistance in performing their job effectively or managing stressful situations [12]. In essence, POS encompasses an officer’s perception of the organization’s recognition, support, and concern for their well-being [31, 32]. Additionally, POS extends to an officer’s belief system regarding the evaluation of organizational policies and procedures in the workplace [11, 33]. Officers use their assessment of POS to gauge the likelihood of the organization acknowledging and valuing their efforts. This assessment also influences the officer’s reciprocal response to the treatment received from the organization [33]. In the context of this research, POS is seen as a means for officers to acquire and apply skills, fostering their development and self-confidence. It forms a reciprocal relationship between learning and the cultivation of enthusiasm and positive energy in the workplace.

#### 2.1.2 Proactive personality

A proactive personality is an inherent trait distinguished by a purposeful tendency to exert intentional influence over situations and the surrounding environment, with the intention of initiating significant transformations [34–36]. An alternative viewpoint regarding this characteristic places emphasis on an individual’s inclination to plan modifications in their environment with minimal regard for situational constraints [37]. Fundamentally, proactive personality pertains to individual qualities that enable officers to consistently endeavor to create and mold a more advantageous milieu. Proactive personalities are characterized by the proposition of innovative ideas and the development of novel approaches to tasks in order to improve the efficiency of organizational functions [38]. Proactive personality, which is defined as "actions taken by officers in advance to influence themselves and/or their surroundings" [39], proves to be an asset to the organization. The implementation of novel concepts and undertakings propelled by officers characterized by proactive dispositions aids in cultivating favorable and constructive transformations within the organizational milieu.

#### 2.1.3 Work engagement

Work engagement is a positive attitude toward one’s job demonstrated by personnel [40]. It includes qualities such as vigor, absorption, and dedication [41]. The presence of vigor in the work environment of officers is associated with increased levels of vitality and psychological resilience [42]. Absorption is demonstrated when officers are completely engrossed in their duties and resistant to interruptions [43]. Dedication is characterized by preparedness to confront challenges, enthusiasm, and motivation. Officers who actively strive to streamline daily operations, complete tasks with greater efficiency, and make more effective use of resources are included in the definition of work engagement [44]. Work engagement is further understood as a concept that encompasses both irrational and logical elements that are associated with the tasks performed and the overall work environment [42, 45]. Low levels of work engagement have been found to have detrimental effects on patient health and compromise the quality of nursing services [44]. With respect to positive organizational behavior and individual mental health, work engagement is considered a positive attribute rather than a deficiency within the field of positive psychology. There is a positive correlation between heightened levels of work engagement and enhanced job performance, which highlights the importance of work engagement in cultivating a favorable business atmosphere.

#### 2.1.4 Voice behavior

Employee voice behavior in officers refers to the proactive expression of opinions or the dissemination of promotional information, contributing innovative suggestions for change [46]. This form of communication places emphasis on constructive challenges, focusing on improvement rather than mere criticism [47, 48]. This type of speaking behavior occurs spontaneously, without explicit encouragement, when an officer harbors an idea or opinion aimed at enhancing a given situation [49]. The direction of voice behavior in officers involves the articulation of opinions or suggestions related to work-related challenges, with the ultimate goal of enhancing organizational efficiency [50]. Furthermore, research indicates a positive correlation between voice behavior and favorable outcomes such as officer job performance and overall organizational effectiveness [38]. In essence, voice behavior plays a pivotal role in organizational success by serving as a catalyst for change and innovation, particularly in challenging times. The introduction of new ideas through employee voice not only facilitates continuous improvement but also contributes to the adaptability and resilience of the organization.

### 2.2 Hypothesis development

#### 2.2.1 Perceived organizational support and voice behavior

According to OST, individuals in the workforce need to comprehend the organization’s level of contribution, significance, and concern for their well-being [19]. The awareness of organizational support is crucial as it fosters stability and a sense of security in the workplace, ultimately leading to positive employee attitudes toward the organization [11]. This positive outlook, in turn, encourages officers to engage in voice behavior, actively offering feedback and suggestions [26].

Perceived organizational support serves as a significant external resource for officers, contributing to emotional recovery and reducing emotional dissonance, thereby facilitating voice behavior. Emotional dissonance, if left unaddressed, can hinder officers’ discretionary, informal, and upward communication, preventing them from expressing their desire to improve existing work processes [51]. Effectively coping with complexity and promoting optimal contributions are additional benefits of perceiving organizational support.

Past studies has established a link between perceived organizational support and employee voice behavior [33, 52]. This connection implies that organizational support plays a role in encouraging individuals to share information and knowledge without reluctance or fear, enabling them to defend their beliefs and those of their team [52]. Particularly noteworthy is the finding that employees who feel the organization cares about them may be more willing to speak up, overcoming personal and career risks associated with voice behavior [33]. Therefore, this study posits the hypothesis that:

H1: Perceived organizational support has a significant and positive influence on voice behavior.

#### 2.2.2 Proactive personality and voice behavior

Individuals with higher levels of proactive personality are more inclined to engage in voice behavior [53], suggesting their significance in the workplace due to their valuable contributions and efforts [18]. Proactive officers have the ability to initiate constructive political discourse, sharing relevant knowledge with fellow organizational members. They excel in effectively communicating their ideas with superiors and leaders, thereby facilitating positive improvements [54].

Fit perceptions, according to TAT [20], are primarily influenced by the interplay between contextual factors and individual differences. Consequently, individual differences, such as proactive personality, play a crucial role in shaping how followers respond, act, and exhibit behaviors like voice behavior.

Numerous studies support the idea that proactive personality significantly contributes to increased voice behavior within organizations [38, 54, 55]. Prior studies specifically found a noteworthy relationship between proactive personality and employee voice behavior, highlighting proactive personality as a key determinant of voicing opinions [38, 52]. Proactive individuals naturally seek opportunities and change [34, 56], making them more likely to participate in activities that demand initiative, such as networking, taking responsibility, and engaging in voice behavior [55]. Essentially, the proactive personality of officers is synonymous with a propensity for voicing ideas, introducing novel approaches to tasks, and conveying innovative suggestions to enhance organizational functions [57]. Consequently, this study hypothesizes that:

H2: Proactive personality has a significant and positive influence on voice behavior.

#### 2.2.3 Work engagement and voice behavior

Numerous studies have underscored the connection between work engagement and the motivation of employees to utilize their voice [26, 58, 59]. The premise is that higher work engagement among officers leads to the perception of voicing opinions as a role that results in increased engagement in voice behavior [26]. Supported by the self-enhancement theory, which posits that individuals aspire to enhance themselves and excel in domains integral to their sense of self [60], engaged employees, having deeply invested their sense of self in their work, are motivated to showcase superior competence and a positive image at the workplace [53].

In addition, expressing promotive and prohibitive voice is a means for engaged employees to demonstrate their outstanding excellence and value by presenting insightful and creative views [53]. Therefore, voice behavior can be seen as a manifestation of self-enhancement. Put differently, the energy and motivation inherent in work engagement contribute to the promotion of voice behavior [61], establishing a clear relationship between work engagement and the expression of voice behavior [62]. Consequently, this study hypothesizes that:

H3: Work engagement has a significant and positive influence on voice behavior.

#### 2.2.4 Perceived organizational support and work engagement

Perceived organizational support among officers instills confidence by signaling that the organization recognizes and values their contributions. This sense of recognition leads to officers’ commitment to the organization’s success and a heightened tendency towards work engagement [63]. The provision of organizational support contributes to fostering positive feelings of security, comfort, and happiness in the workplace, thereby enhancing officers’ physical and mental relationship with their work, ultimately resulting in elevated work engagement [26].

According to social exchange theory [64], the reciprocal effect of perceived organizational support influences officers’ emotional attitudes towards the organization. This, in turn, prompts officers to offer work resources, initiating motivational processes that culminate in work-related effort [19], with work engagement being one of the potential outcomes [65]. This perspective is further supported by previous study, which argue that individuals who feel valued and respected by the organization are likely to reciprocate with higher levels of positive work outcomes, including engagement, commitment, and performance [66].

Several studies reinforce the significant relationship between perceived organizational support and work engagement [65, 67, 68]. Therefore, this study posits the hypothesis that:

H4: Perceived organizational support has a significant and positive influence on work engagement.

#### 2.2.5 Proactive personality and work engagement

Numerous previous studies have consistently identified a positive relationship between proactive personality and work engagement [15, 18, 24, 69]. This suggests that individuals characterized by a proactive personality tend to introduce innovative ideas in the workplace and exhibit high levels of absorption, enthusiasm, and dedication to their work [15]. Additionally, those with a proactive personality are actively engaged in their work, indicating a significant connection between proactive personality and work engagement [24].

In essence, employees with a robust proactive personality are more likely to fully invest themselves in their work, performing tasks at their maximum potential and demonstrating a high level of absorption in their work, ultimately leading to elevated levels of work engagement [69]. Therefore, this study posits the hypothesis that:

H5: Proactive personality has a significant and positive influence on work engagement.

#### 2.2.6 Work engagement mediation

The previous study reveals that work engagement serves as a mediator in the relationship between perceived organizational support and voice behavior [26]. This implies that officers are likely to engage in voice behavior when they invest themselves continuously in their work roles, and this inclination is facilitated by the presence of supportive organizational structures [53]. Officers who are engaged in their work are more likely to influence voice behavior when they perceive voicing as safe and effective [70]. Conversely, while no study has specifically explored the mediating role of work engagement between proactive personality and voice behavior, officers with a proactive personality are generally more motivated to undertake enjoyable tasks and avoid personal risks [38]. According to past study [51], alignment between the emotions officers feel and express can generate greater power, increasing work engagement and subsequently leading to more extra-role behaviors, such as voice behavior.

Work engagement is a powerful and dedicated mechanism linking employees to their work tasks with a clear identification of their roles [24]. The theoretical arguments presented thus far suggest that perceived organizational support and proactive personality influence employee voice behavior through the mediating role of work engagement. This conceptual framework aligns with various studies demonstrating work engagement as a mediator between perceived organizational support with various work attitudes and behaviors crucial to organizations, including job performance [71], organizational citizenship behavior [72, 73], proactive behavior [74], employee creativity [75], and intention to stay [76]. Similarly, work engagement acts as a mediator between proactive personality and essential work behaviors like job performance, innovative work behavior, and creative performance [15, 24, 69]. Therefore, this study hypothesizes that:

H6: Work engagement significantly mediates the positive influence of perceived organizational support on voice behavior.
H7: Work engagement significantly mediates the positive influence of proactive personality on voice behavior.

**Fig 1:**
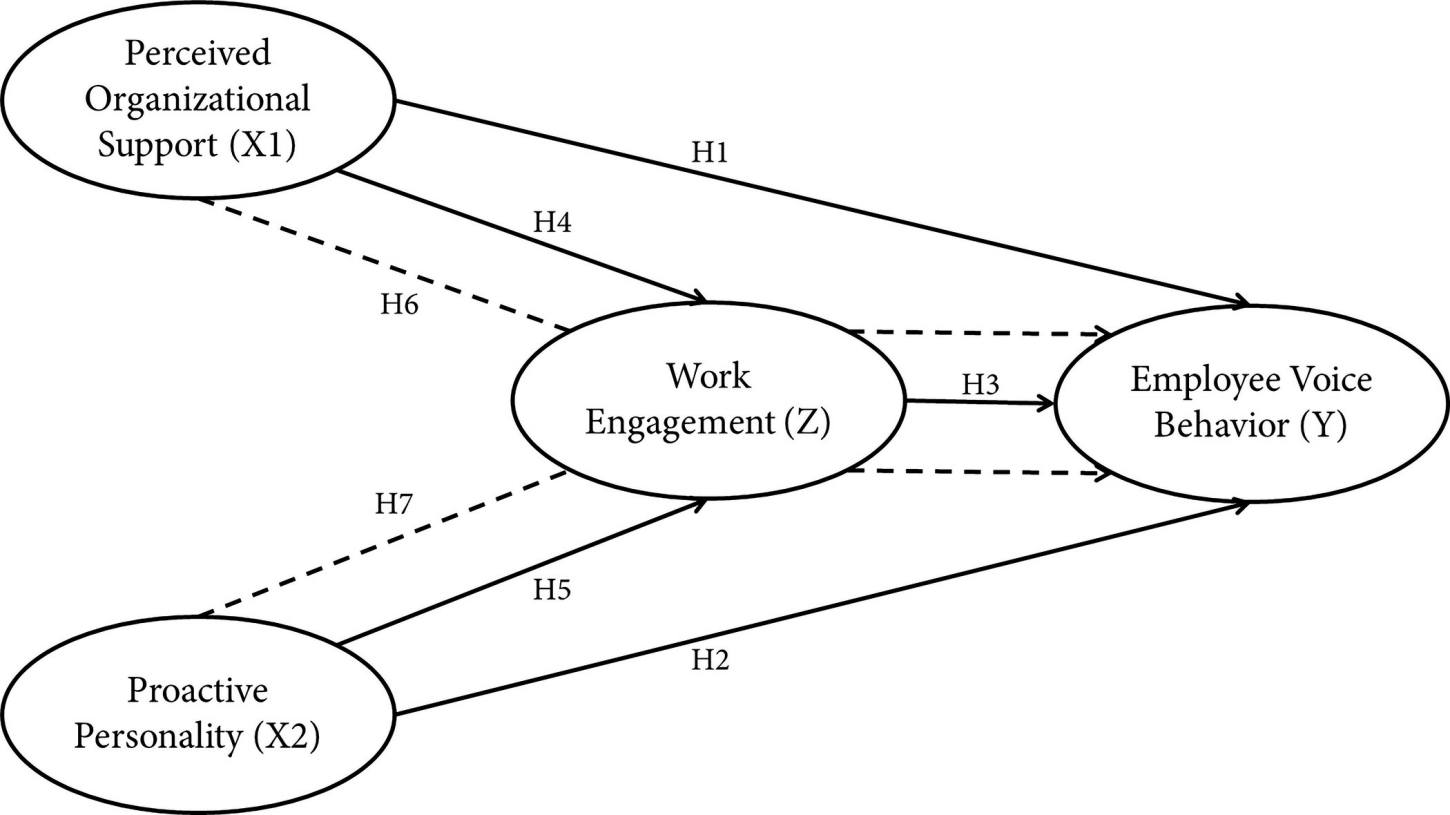
Conceptual framework.

All hypotheses are illustrated in the following framework (Fig 1).

## 3. Method

### 3.1 Data collection procedures

The research data collection involved the distribution of online questionnaires accessible via computers or smartphones to correctional health service officers in Indonesia. The questionnaire data collection method utilized the time-lagged approach, where the questionnaire was distributed twice with a 30-day interval, and a waiting period of 7 days for each distribution stage, totaling 44 days. In the first questionnaire distribution stage (T1), which was conducted on 10 October 2022–4 November 2022, respondents provided information on their identity and answered 18 items related to perceived organizational support and proactive personality. The second questionnaire distribution stage (T2), which was done on 5–30 December 2022, included 10 identity-related items and 23 items related to work engagement and employee voice behavior. Respondents spent approximately 5–10 minutes completing each questionnaire. Both questionnaires included information and questions addressing ethical considerations, such as identity confidentiality and consent to participate in the study.

The initial distribution (T1) yielded data from 363 respondents, while the second distribution (T2) had 331 respondents. Subsequently, an examination was conducted to identify respondents who completed both questionnaires, resulting in 287 eligible respondents for testing. Analysis of respondent characteristics indicated a majority of women (50.87%), aged 31–40 years (37.63%), with over 15 years of work experience (37.63%), and holding a diploma education level (27.87%). The full demographic distribution can be found in Table 1.

**Table 1:** Characteristics of respondents.

Demography	Total	%
**Gender**		
Man	141	49.13
Woman	146	50.87
**Age**		
20–30 years old	70	24.39
31–40 years old	108	37.63
41–50 years old	64	22.30
>50 years old	45	15.68
**Tenure**		
< 1 Year	38	13.24
1–3 Years	16	5.57
4–6 Years	41	14.29
7-9Years	6	2.09
10–12 Years	43	14.98
13–15 Years	35	12.20
> 15 Years	108	37.63
**Working area**		
Bali	17	5.92
East Java	33	11.50
West Java	30	10.45
Central Java	17	5.92
Etc	190	66.20
**Education**		
Diploma	80	27.87
Bachelor	152	52.96
Master	20	6.97
Senior High	35	12.20
**Position/Title**		
Doctor	22	7.67
Nurse	120	41.81
Midwife	3	1.05
Health Staff	142	49.48
**Institution/Facility**		
Correctional institutions	181	63.07
Detention centre	76	26.48
Narcotics correctional units	20	6.97
Women’s correctional units	10	3.48

Since the present investigation excludes vulnerable populations and does not involve specific interventions or treatments for respondents, the Research and Publication Center (RPC) at the Faculty of Economics and Business, Universitas Airlangga, has concluded that ethical approval is not required. Before responding to the questionnaire, participants granted written consent, receiving assurance that their information would be handled confidentially and utilized exclusively for research purposes. The RPC has duly verified and validated this obtained consent.

### 3.2 Measurement

The independent variables used in this research are *perceived organizational support* and proactive personality, then the mediating variable used is work engagement, whereas in this research the dependent variable used is voice behavior (see Fig 1). This research measures perceived organizational support using eight unidimensional items [77]. Next, proactive personality uses ten items [78]. Work engagement was measured using the work and well-being survey (UWES) with 17 items divided into the dimensions of vigor, dedication and absorption [40], while voice behavior was measured with six items [47]. All measurement items use a 5-point Likert scale (strongly disagree—strongly agree).

### 3.3. Data analysis technique

Partial Least Squares—Structural Equation Modeling (PLS-SEM) was used in this study using the SmartPLS 4 program for analysis. A variance-based statistical technique called PLS-SEM can assess the measurement model and then the structural model at the same time [79]. This technique is said to be better for regression analysis when assessing mediation, this study adopted it [80]. In addition, PLS-SEM fits well into the current research environment, which is concerned not only with testing hypothetical models but also with obtaining managerial recommendations [80–82]. In addition, PLS-SEM has the causal-predictive power to achieve a balance between the research objectives of building explanations and revealing predictions [81].

Model testing in this study uses the hierarchical component model or more commonly known as the repeated indicator approach [83], to ensure that high-order constructs or dimensions in the work engagement variables (vigor, dedication, and absorption) are also tested. Apart from that, this avoids misspecification and obsolete models such as only modeling and lower-order constructs [84]. By making thorough and careful efforts in specification, estimation, and validation of research models which contain higher-order constructs [84, 85].

Technically reporting the results in this study consists of measurement model assessment and structural model assessment [86]. In the measurement model assessment, the results of testing indicator loadings, internal consistency reliability (Cronbach’s alpha and composite reliability), convergent validity or average variance extracted (AVE), and discriminant validity consisting of fornell-lacker criterion and heterotrait-monotrait (HTMT) ratio are reported. Then the structural model assessment reported the coefficient of determination (R^2^), the blindfolding-based cross-validated redundancy measure (Q^2^), and effect size (ƒ^2^), the significance of the path coefficient. In addition, the structural model test was run using 10,000 subsamples bootstrapping on a one-tail basis to adjust and offer a powerful approach to obtain more robust results [87] and fit the theoretical foundations of the direction of the relationship in the model.

## 4. Results and discussion

### 4.1 Measurement model assessment

The measurement model results show that the indicators loadings, internal consistency reliability, convergent validity, and discriminant validity are met (see Table 2). Based on the loadings indicator, it shows that all items from perceived organizational support, proactive personality, work engagement (low-order construct and high-order construct), and employee voice behavior show that they are worthy of being maintained (>0.4), so that all items are not eliminated [79]. Then the internal consistency reliability results show the overall construct in the satisfactory model in Cronbach’s alpha and composite reliability (>0.7) [79]. Next, convergent validity which is reviewed from AVE (0.581–0.854) produces a value above >0.5, so that all construct explains more than half of the variance of its indicators [79].

**Table 2:** Measurement models.

Construct	Indicators		First Stage				
		Mean	Outer Loading Low-Order	High-Order Outer Loadings	Cronbach’s Alpha	Composite Reliability	AVE
**Perceived Organizational Support**	POS1	4,258	0.803		0.922	0.923	0.647
	POS2^®^	4,338	0.841				
	POS3^®^	4,209	0.827				
	POS4	4,185	0.789				
	POS5^®^	4,268	0.825				
	POS6	4,199	0.761				
	POS7^®^	4,230	0.804				
	POS8	4,185	0.783				
**Proactive Personality**	PP1	4,530	0.722		0.917	0.926	0.581
	PP2	4,443	0.784				
	PP3	4,355	0.810				
	PP4	4,456	0.760				
	PP5	4,251	0.814				
	PP6	3,307	0.493				
	PP7	3,812	0.806				
	PP8	4,436	0.787				
	PP9	3,993	0.786				
	PP10	3,861	0.803				
**Work Engagement**					0.970	0.972	0.678
**Vigor**	VI1	4,467	0.860	0.883	0.920	0.924	0.718
	VI2	4,519	0.839	0.859			
	VI3	4,383	0.806	0.864			
	VI4	4,272	0.769	0.845			
	VI5	4,394	0.861	0.898			
	VI6	4,303	0.717	0.724			
**Dedication**	DE1	4,571	0.904	0.944	0.961	0.962	0.864
	DE2	4,575	0.916	0.955			
	DE3	4,557	0.911	0.948			
	DE4	4,669	0.845	0.911			
	DE5	4,523	0.826	0.888			
**Absorption**	AB1	4,554	0.869	0.857	0.902	0.912	0.673
	AB2	3,899	0.665	0.753			
	AB3	4,418	0.861	0.891			
	AB4	4,551	0.854	0.870			
	AB5	4,038	0.651	0.708			
	AB6	4,247	0.787	0.829			
**Employee Voice Behavior**	EVB1	3,784	0.904		0.937	0.947	0.762
	EVB2	3,749	0.896				
	EVB3	3,812	0.866				
	EVB4	3,958	0.928				
	EVB5	3,355	0.749				
	EVB6	3,763	0.883				

* Notes:

^®^ = Reverse Item

Discriminant validity testing shows that the Heterotrait-Monotrait (HTMT) ratio is below the threshold of 0.9 (see Table 3), so it is suitable for further analysis [88]. In addition, the results can imply that each construct is unique and captures phenomena not represented by other constructs in the model [79].

**Table 3:** Discriminant validity.

Fornel-Lacker Criterion					Heterotrait-Monotrait Ratio (HTMT)				
	EVB	POST	PP	WE		EVB	POST	PP	WE
EVB	0.873				EVB				
POST	0.293	0.805			POST	0.310			
PP	0.595	0.327	0.762		PP	0.631	0.357		
WE	0.549	0.437	0.600	0.824	WE	0.576	0.459	0.636	

### 4.2 Structural model assessment

In testing the structural model, the results obtained were explained variance (R^2^), effect size (ƒ^2^), and predictive power (Q^2^) from the two dependent variables in the model tested (see Table 4). These metrics signify the degree to which exogenous variables factors can explain and predict endogenous variables (employee voice behavior) [79], thus statistically supports the findings of this study. The acceptance of R^2^ is contingent upon the academic discipline, with the lowest acceptance rates observed in fields focusing on human behavior due to its inherent unpredictability compared to phenomena studied in the natural sciences. In the realm of social sciences, an R^2^ value of 0.1 is deemed acceptable, indicating a satisfactory level of explained variance, while a value of 0.20 is considered high [89]. The results of employee voice behavior (R^2^ = 0.413, Q^2^ = 0.307) and work engagement (R^2^ = 0.425, Q^2^ = 0.282) can be said to be moderate in explained variance and medium in predictive power [79, 81]. Furthermore, the results of the effect size (ƒ^2^) show that the five direct effects in the model vary between small, medium, and large effects [79]. The direct influence between perceived organizational support and employee voice behavior shows a small effect (0.02–0.14). Meanwhile, the direct influence of perceived organizational support on work engagement, proactive personality on employee voice behavior, and work engagement on employee voice behavior show a medium effect (0.15–0.34). Furthermore, large effects (>0.35) were found in the direct influence of proactive personality on employee voice behavior.

**Table 4:** Structural model testing.

Dependent Variable	R2	F2			Q2
		POST	PP	WE	
**EVB**	0.413	0.001	0.183	0.081	0.307
**WE**	0.425	0.113	0.407		0.282

**Fig 2:**
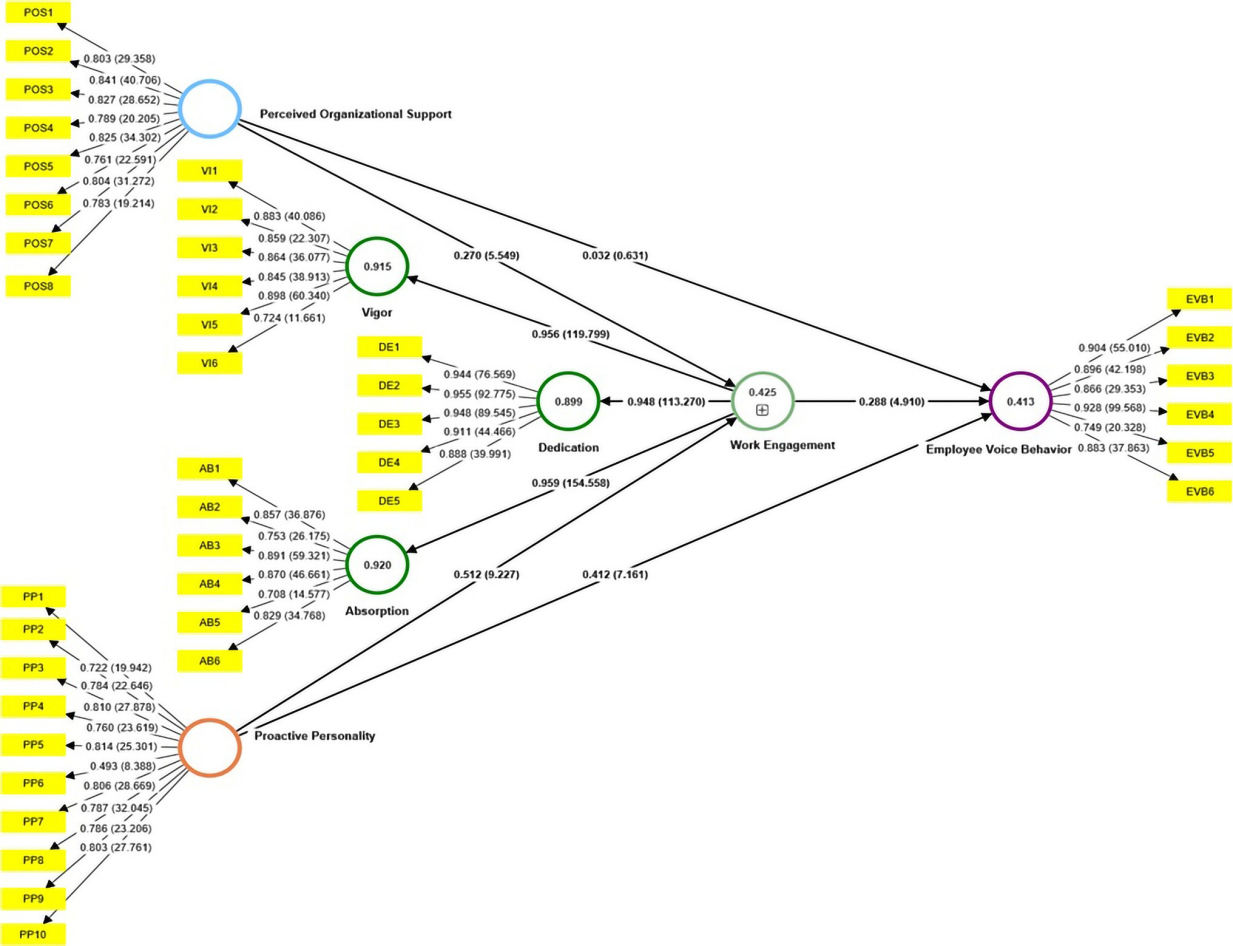
Structural model test results.

The subsequent analysis pertained to the path coefficient’s significance. The findings indicated that several of the hypotheses formulated in this research were statistically significant and exhibited a positive correlation (refer to Table 5 and Fig 2). The findings regarding the direct impact of perceived organizational support on employee vocal behavior (H1) indicate a statistically insignificant positive influence (β = 0.032, t = 0.631, p > 0.05). The findings from the analysis of the direct impact of proactive personality on employee voice behavior (H2) indicated a statistically significant and positive relationship (β = 0.412, t = 7.161, p<0.05). The test outcomes pertaining to the direct impact of work engagement on employee vocal behavior (H3) indicate a statistically significant and positive relationship (β = 0.288, t = 4.910, p<0.05). Furthermore, it is noteworthy that the direct impact of perceived organizational support on work engagement (H4) is also statistically significant and positive (β = 0.270, t = 5.549, p<0.05). Proactive personality exhibited the most substantial and statistically significant positive direct effect (β = 0.512, t = 9.227, p<0.05) on work engagement (H5).

**Table 5:** Direct and indirect effect testing.

Path	Original Sample (β)	Sample Mean	Standard Deviation	T Statistics	P Values	Hypothesis
**POS → EVB**	0.032	0.035	0.051	0.631	0.264	H1 Rejected
**PP → EVB**	0.412	0.412	0.057	7,161	0,000	H2 Supported
**WE → EVB**	0.288	0.288	0.059	4,910	0,000	H3 Supported
**POS → WE**	0.270	0.271	0.049	5,549	0,000	H4 Supported
**PP → WE**	0.512	0.515	0.055	9,227	0,000	H5 Supported
**POS → WE → EVB**	0.078	0.078	0.021	3,716	0,000	H6 Supported Full Mediation
**PP → WE → EVB**	0.147	0.148	0.036	4,117	0,000	H7 Supported Partial Mediation

Note:

POS: Perceived Organizational Support WE: Work Engagement

PP: Proactive Personality EVB: Employee Voice Behavior

*: significant at the 0.05 level ns: not significant at the 0.05 level

The results of the indirect influence test show that work engagement is proven to mediate the influence of perceived organizational support on employee voice behavior (H6). The results of this mediation test are significant (β = 0.078, t = 3.176, p<0.05) and have fully mediation properties, so it can be said that perceived organizational support can only increase (enhance) *employee voice behavior* through work engagement. Furthermore, the results of the indirect influence show that the mediating role of work engagement in the influence of proactive personality on employee voice behavior (H7) is proven to be significant (β = 0.147, t = 4.117, p<0.05) with the nature of partial mediation. These results show that the influence of proactive personality on increase (enhance) employee voice behavior can occur in direct influence or indirect influence through work engagement.

### 4.3 Discussion

The findings of this study indicated that perceived organizational support did not have a significant impact on employee voice behavior, diverging from prior research that suggested a direct influence [33, 52]. Contrarily, the study revealed a complete mediating effect of work engagement on the relationship between perceived support and employee behavior. This suggests that the influence of perceived support on employee voice behavior is solely channeled through work engagement. These results align with previous study [26], reinforcing the notion that when both correctional institutions and detention centers acknowledge the additional efforts of health workers, it fosters dedication, instills a sense of inspiration in their work, and subsequently cultivates a willingness to express voice behavior, as their opinions are perceived as valuable.

Furthermore, the study demonstrated that proactive personality exerted a positive direct effect on employee voice behavior, consistent with previous research findings [38, 54, 55]. These outcomes support the TAT [20], emphasizing that fit perceptions predominantly result from the interplay between contextual factors and individual differences. The results underscore that individuals with proactive personalities are inclined to respond to their surroundings by expressing their ideas and thoughts through voice behavior. Additionally, the findings highlight that when health workers are confident in realizing their aspirations, they are more likely to make concerted efforts to communicate their desires, believing it can benefit their organization.

Subsequently, the outcomes of this study demonstrated that work engagement exerted a significant and positive direct influence on voice behavior. These results align with several preceding studies that yielded similar conclusions [26, 58, 59]. These findings provide further support for the Self-enhancement theory [60], asserting that individuals strive for self-improvement and excellence in their areas of proficiency. This inclination encourages individuals to confidently express opinions, particularly in areas they have mastered, contributing to desired improvements. Moreover, the findings suggest that healthcare workers who exhibit high dedication and serve as inspirers in the correctional environment are more likely to engage in voice behavior, perceiving their opinions as highly beneficial to the organization.

Additionally, the test results revealed a direct positive effect of perceived organizational support on work engagement. This outcome supports the reciprocal effect outlined by social exchange theory [64], where organizational support shapes an officer’s emotional attitude toward the organization, prompting the officer to provide work resources. This, in turn, triggers a motivational process leading to work-related effort, as indicated by high work engagement. Furthermore, these study findings align with several prior research results [65, 67, 68]. The positive impact of perceived organizational support on work engagement implies that healthcare workers receive acknowledgment and support for their hard work from the organization, fostering reciprocal high dedication from the staff.

The correlation between work engagement and proactive personality exhibits the most significant direct positive effect. This result is consistent with the findings of a number of empirical investigations that have examined the relationship between proactive personality and work engagement [15, 18, 24, 69]. The robust direct effect indicates that organizational factors, such as organizational support, have a lesser impact on work engagement than individual factors, such as personality characteristics. This discovery emphasizes that healthcare professionals who exhibit self-assurance and perseverance in attaining their goals foster a psychological state marked by enthusiasm, dedication, and intense effort.

In conclusion, the findings of the research align with the anticipated positive correlations between work engagement and voice behavior, as well as proactive personality and work engagement. The results demonstrate that work engagement partially mediates the relationship between proactive personality and voice behavior. This finding underscores the significance of work engagement, which includes both irrational and rational aspects related to work and the overall work experience [45]. It acts as a channel through which proactive employees can voice their thoughts and suggestions regarding ways to enhance the organization. As a result, the findings indicate that healthcare workers who take initiative are more likely to demonstrate significant levels of work engagement. This, in turn, encourages them to participate in voice behavior, which they perceive as advantageous for their own development and for facilitating positive transformations within their organization.

## 5. Conclusion

The test results of this study predict that the perceived organizational support of health care workers in correctional institutions and detention centers cannot directly influence these officers to display voice behavior in the workplace. The perceived organizational support of the officers is predicted to be able to produce voice behavior only through the mediating effect of the work engagement of these officers.

In addition, the results of this study predict that health care workers who have a proactive personality tend to voice behavior both directly and depending on the officer’s work engagement. Then the results of this study also predict that perceived organizational support and proactive personality of health care workers can encourage work engagement in the workplace. The results of this study predict that work engagement partially mediates the effect of proactive personality on voice behavior significantly. Thus, work engagement plays a key role as absolute mediation in the proposed model.

## 6. Implications

### 6.1 Theoretical implications

Based on the existing literature, the results of this study have several theoretical contributions. First, this study shows the interplay between proactive personality, perceived organizational support, and work engagement on constructive behavior for the organization such as voice behavior. The results of this study demonstrate that voice behavior will be displayed by proactive officers either directly or indirectly with the mediation of work engagement. This is relevant to TAT [20], implying that the personality in a person will be activated in the organization through attitudes and behaviors that align with that personality. Second, this study demonstrates that perceived organizational support cannot have an effect on increasing officers’ voice behavior. Second, this study demonstrates that perceived organizational support cannot have an effect on increasing officers’ voice behavior. However, this effect will occur through the mediation mechanism of work engagement. This shows that work engagement is a key link to the organizational support that is sought in building voice behavior in the context of constructive improvement. Third, although this study predicts that perceived organizational support cannot influence voice behavior, the findings of this study show that perceived organizational support affects work engagement. This is relevant to OST [19], where individual awareness of organizational support is able to provide stability and safety in the workplace so that the main significant result of such support is the employee’s positive attitude towards the organization [11], as work engagement is displayed by officers in the workplace.

### 6.2 Managerial implications

The insights gained from the diverse causal predictions in this study offer valuable guidance and recommendations for managers and organizations, particularly within the context of correctional institutions and detention centers. Firstly, given the persistent challenges faced by correctional facilities in terms of health facilities and personnel shortages, it is imperative for health workers to receive ample organizational support. This support aims to enhance staff engagement, fostering optimal performance to improve services. The subsequent benefits include a reduction in morbidity and mortality rates, contributing to the establishment of a healthier environment within correctional institutions and jails. Secondly, the study underscores the pivotal role of proactive personality traits among health workers in correctional units and detention center. Consequently, organizations can play a crucial role by empowering and instilling proactive values in their workforce. This approach fosters constructive progress through the introduction of innovative ideas from officers. In essence, correctional institutions and detention centers can facilitate bottom-up changes, generating positive and sustainable impacts. Thirdly, the study findings offer recommendations for correctional institutions and detention center management to concurrently enhance support for health service workers and encourage proactive behavior. This dual approach aims to cultivate engagement among officers, enabling them to effectively carry out health duties in rehabilitative, curative, preventive, and promotive aspects. By addressing both organizational support and proactive attitudes, correctional institutions and detention center management can foster an environment conducive to optimal healthcare delivery. Lastly, this study underscores the significance of proactive personality in fostering employee voice behavior, which holds potential for fostering positive transformations within health services in correctional facilities, encompassing rehabilitative, curative, preventive, and promotive dimensions. Consequently, managers within correctional institutions should integrate proactive personality assessments into the selection process for health workers.

## 7. Limitations and directions for future research

This study has both strengths and limitations. The main strength of this study is its two-wave and time-lagged research design. Temporal segregation of data was done using two waves study design to collect data for perceived organizational support and proactive personality at time 1 (T1) and work engagement and employee voice behavior at time 2 (T2). This strategy helped us in minimizing the concerns regarding common method bias [90]. To improve the results’ accuracy, the data at T1 and T2 were collected from the same employees and matched time-lagged responses.

However, this study is not without limitations. One of the weaknesses of this study comes from the sample. As such, the results of this study cannot be generalized to organizations such as the private sector. Given these limitations, future research should focus on replicating this study across different public sector and industrial contexts using a cross-lagged or diary study research design. The conduct of a longitudinal study design would help to address the causality issues present in our study. Future studies should also examine other mediating and moderating mechanisms to understand the processes and conditions. For example, political skills, job constrains, and emotional regulation can be considered as potential moderators in the proactive personality–employee voice behavior relationship.
